# Mid- to long-term clinical and radiological results of anatomic total shoulder arthroplasty in patients with B2 glenoids

**DOI:** 10.1016/j.jseint.2023.01.006

**Published:** 2023-02-11

**Authors:** Stephanie Hinse, Torsten Pastor, Anita Hasler, Lukas Ernstbrunner, Karl Wieser, Christian Gerber

**Affiliations:** Department of Orthopaedics, Balgrist University Hospital, Zurich, Switzerland

**Keywords:** Mid-term results, Anatomical total shoulder arthroplasty, Glenohumeral osteoarthritis B2 glenoid, Posterior glenoid deficiency, Humeral subluxation

## Abstract

**Background:**

Eccentric biconcave (B2) glenoid erosion in primary glenohumeral arthritis is common. There are serious concerns regarding the longevity of fixation of cemented glenoids if anatomic total shoulder arthroplasties (aTSAs) are used in B2 glenoid. The purpose of this study is to analyze the mid- to long-term results of aTSA with B2 glenoids.

**Methods:**

This is a retrospective study of a single center experience. Thirty patients (32 shoulders) at an average of 9.2 years (range, 5.0-16.6, ±3.2) after primary TSA were evaluated. Clinical and radiographic outcomes were analyzed.

**Results:**

The mean preoperative intermediate glenoid version was −14° ± 7° (range, −2° to −29°) and the mean humeral subluxation according to the plane of the scapula was 67% ± 9% (range, 49%-87%). There was a significant improvement for all the postoperative clinical outcome parameters including the mean absolute and relative Constant Score, subjective shoulder value, active elevation, external rotation, abduction, internal rotation, pain scores, and strength (*P* < .001). The complication rate was 15.6% and the revision rate was 12.5% at a mean follow-up of 9.2 years (range, 5.0-16.6, ±3.2). The estimated survivorship without revision was 94% at 5 years and 85% at 10 years (12.1-14.7 years). The survival rate without advanced glenoid component loosening (defined as Lazarus grade ≥ 4 or modified Molé scores ≥ 6) was 91% at 5 years and 84% at 10 years (12.2-15.8 years).

**Conclusion:**

In this case series, aTSA with asymmetric reaming for the treatment of shoulder osteoarthritis with milder forms of B2 glenoid is a viable option with good to excellent clinical results and an 85% prosthetic survivorship at 10 years.

Eccentric biconcave (B2) glenoid erosion in glenohumeral arthritis is common and presents challenges due to posterior glenoid bone loss, static posterior humeral head subluxation, and eccentric loading.^28^ The surgical treatment is challenging and controversial as none of the treatment options have been proven to be superior to others.^16^ Several surgical techniques have been described for the treatment of this condition and included hemiarthroplasty, anatomic total shoulder arthroplasty (aTSA) with asymmetric reaming of the high side anteriorly, TSA with posterior glenoid bone grafting or posteriorly augmented glenoid component, and reverse TSA (rTSA).^16^

aTSA is associated with an increased failure rate in the eccentrically eroded B2 glenoids and alarming reports have been published on their high risk of early complications such as glenoid loosening and dislocations.^13^^,^^29^ At a mean follow-up of 77 months, Walch et al^29^ reported a 20.6% of glenoid loosening and a rate of revision of 16.3% in 92 aTSA (75 patients) in B2 glenoids. To our knowledge, only short- and mid-term results of aTSA with asymmetric reaming in B2 glenoid have been published for cemented polyethylene glenoid components and the longest series had a mean follow-up of 77 months.^2^^,^^6^^,^^19^^,^^29^

The purpose of this study was to report the mid- to long-term clinical and radiographic findings in patients with glenohumeral osteoarthritis and eccentric B2 glenoid deformity treated with aTSA. The main hypothesis was that cemented glenoids implanted in the presence of a preoperative B2 glenoid show early radiographic signs of loosening leading to a high revision rate associated with deterioration of the clinical outcome.

## Materials and methods

This retrospective case series was approved by the ethical committee responsible for our institution and an additional written consent was obtained from all patients.

### Patients

Between 2002 and 2013, 233 patients (262 shoulders) had an aTSA performed by the senior author (C.G.) or under his direct supervision at our institution. To identify B2 glenoids, the preoperative imaging including shoulder radiographs and computed tomography (CT) scans were reviewed by 2 independent observers (A.H., S.H.). Final selection was approved by a general agreement (C.G., A.H., and S.H.) on the CT-based Classification. Exclusion criteria included patients treated with metal backed glenoid, previous arthroplasty surgery, and aTSA done before 2002 because a different glenoid implant had been used. A preoperative B2 glenoid deformity was identified in 47 shoulders (45 patients) and further analyzed. All patients were operated on after failed conservative treatment with the indication to treat painful glenohumeral osteoarthritis with a biconcave B2 glenoid in the presence of an intact rotator cuff. All patients were contacted for clinical and radiological examination and a minimum 5 years follow-up was needed for the purpose of this study. At the time of final follow-up, 12 patients (25.5%) had died from unrelated causes without a complete follow-up of 5 years. One patient could not be reached and 2 patients refused to come back for a follow-up but verbally confirmed that they did not have any shoulder revision since their primary TSA and accepted to answer a telephone questionnaire including the subjective shoulder value (SSV) and overall satisfaction of the postoperative results that were rated as “excellent,” “good,” “fair,” or “unsatisfactory”. Both of these patients had a SSV of 90% and evaluated their results as “excellent”.

**Figure 1:**
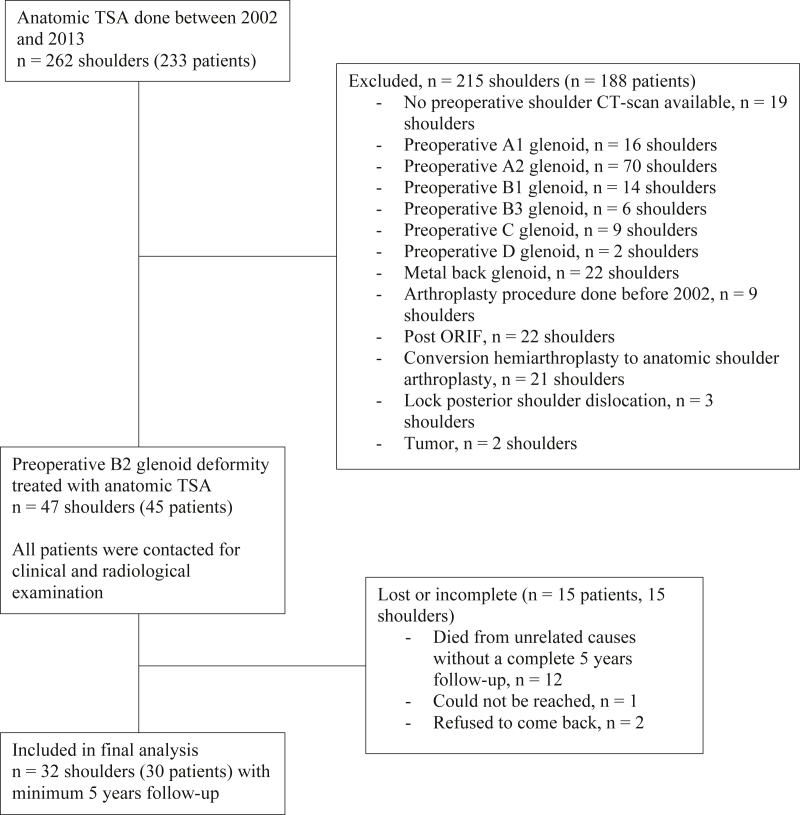
Flowchart of patient selection. *ORIF*, Open reduction internal fixation; *TSA*, total shoulder arthroplasty; *CT*, computed tomography; *ORIF*, open reduction internal fixation.

A total of 32 shoulders in 30 patients were left for final analysis (Fig. 1) and characteristics are shown in Table I.

**Table I:** Demographics of the 30 patients (32 shoulders) who underwent anatomic TSA in B2 glenoids.

Epidemiology	Absolute value	Percentage
Number of patients	30	N/A
Number of shoulders	32	N/A
Mean age at surgery, yrs (range; SD)	65.1 (36.5-77.1; ±9.0)	N/A
Mean follow-up, yrs (range; SD)	9.2 (5.0-16.6; ±2.8)	N/A
Male, n	14	47
Female, n	16	53
Dominant side involvement, n	19	59
Mean body mass index	26.2	N/A
Primary procedure, n	26	81
Previous non-arthroplasty procedure, n	6	19

*SD*, Standard deviation; *N*, number of shoulders; *TSA*, total shoulder arthroplasty.

### Surgical technique

All patients were treated with an Anatomical Shoulder Prosthesis (Zimmer-Biomet, Warsaw, IN, USA) with a cemented all-polyethylene glenoid component (27 pegged and 5 keeled) through a deltopectoral approach and a standard technique for all cases.^7^^,^^18^ The glenoid retroversion was analyzed on the preoperative CT scan. Normal glenoid retroversion was defined as 0° to −12° of retroversion^21^ and an eccentric reaming technique was used with a maximum of 10° of correction to restore the glenoid version to as close to physiologic values as possible.^9^ Reaming was done so as to definitely not destroy the subchondral bone plate in the posterior half to two-thirds of the glenoid surface. The goal was to achieve an arbitrary minimum of 75% of glenoid support for the glenoid implant. A low viscosity bone cement with antibiotics (Palacos-LV + G; Zimmer Biomet, Warsaw, IN, USA) was pressurized in the glenoid bone and cement was also applied on the back of the glenoid implant.^18^ No patient had a posterior capsular plication. The humeral component was cemented in 14 shoulders (44%).

### Clinical and radiographic assessment

Preoperative and postoperative clinical assessments were done by an independent examiner who had not operated on the patients. The Constant-Murley Score (aCS) was measured and the relative CS (rCS) was evaluated as a percentage of the age and sex-matched normal score.^3^ All the active and passive ranges of motion were measured with a handheld goniometer while the patient was seated and the abduction strength was recorded with a validated electronic dynamometer (Isobex; Cursor, Bern, Switzerland). The SSV was collected.^8^

**Figure 2:**
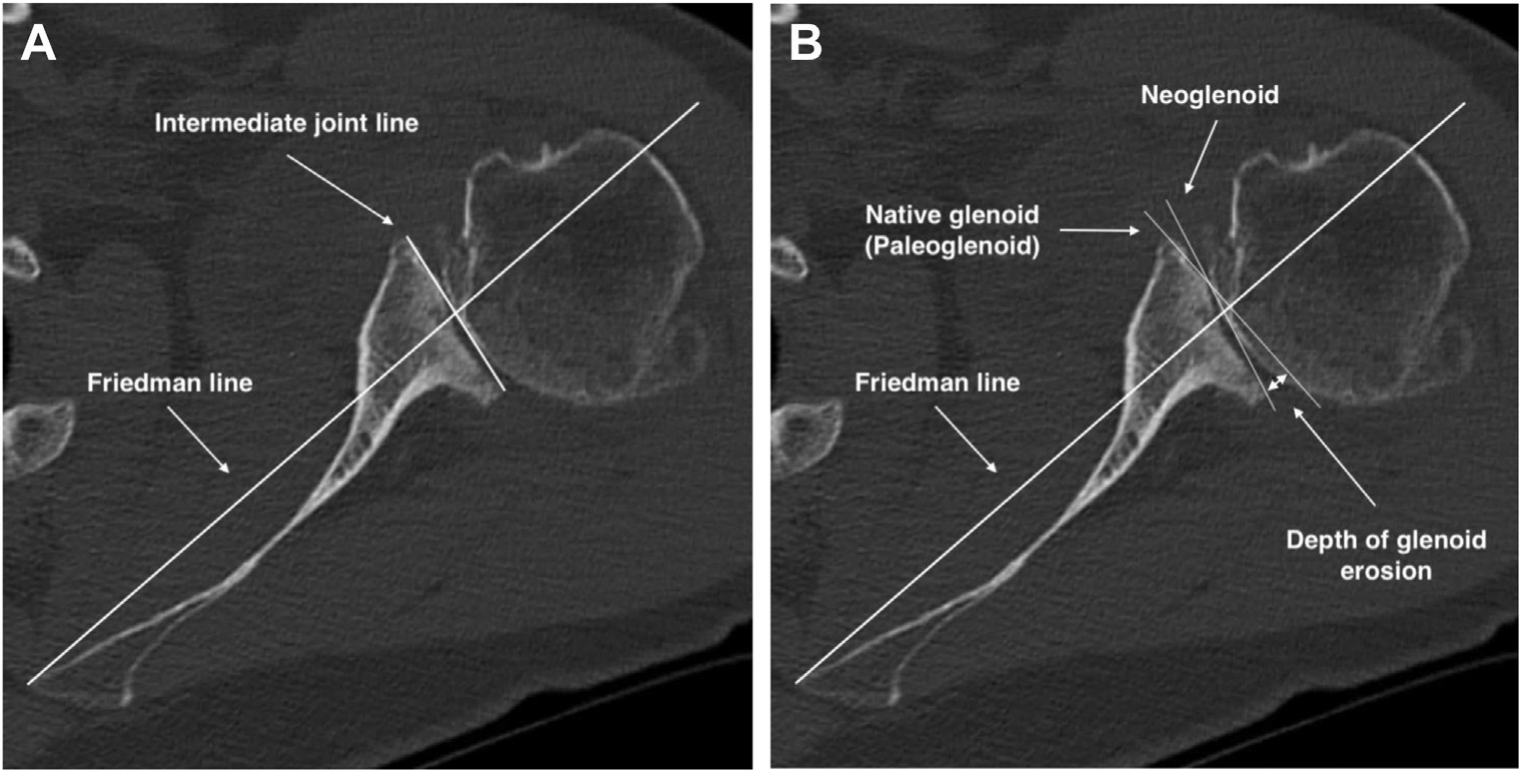
Glenoid retroversion measurements. **(A)** Intermediate glenoid retroversion according to the Friedman line. **(B)** Paleoglenoid and neoglenoid retroversion according to the Friedman line. The depth of posterior glenoid erosion is measured by establishing the perpendicular distance in millimeter between the paleoglenoid line and the posterior border of the eroded glenoid.

**Figure 3:**
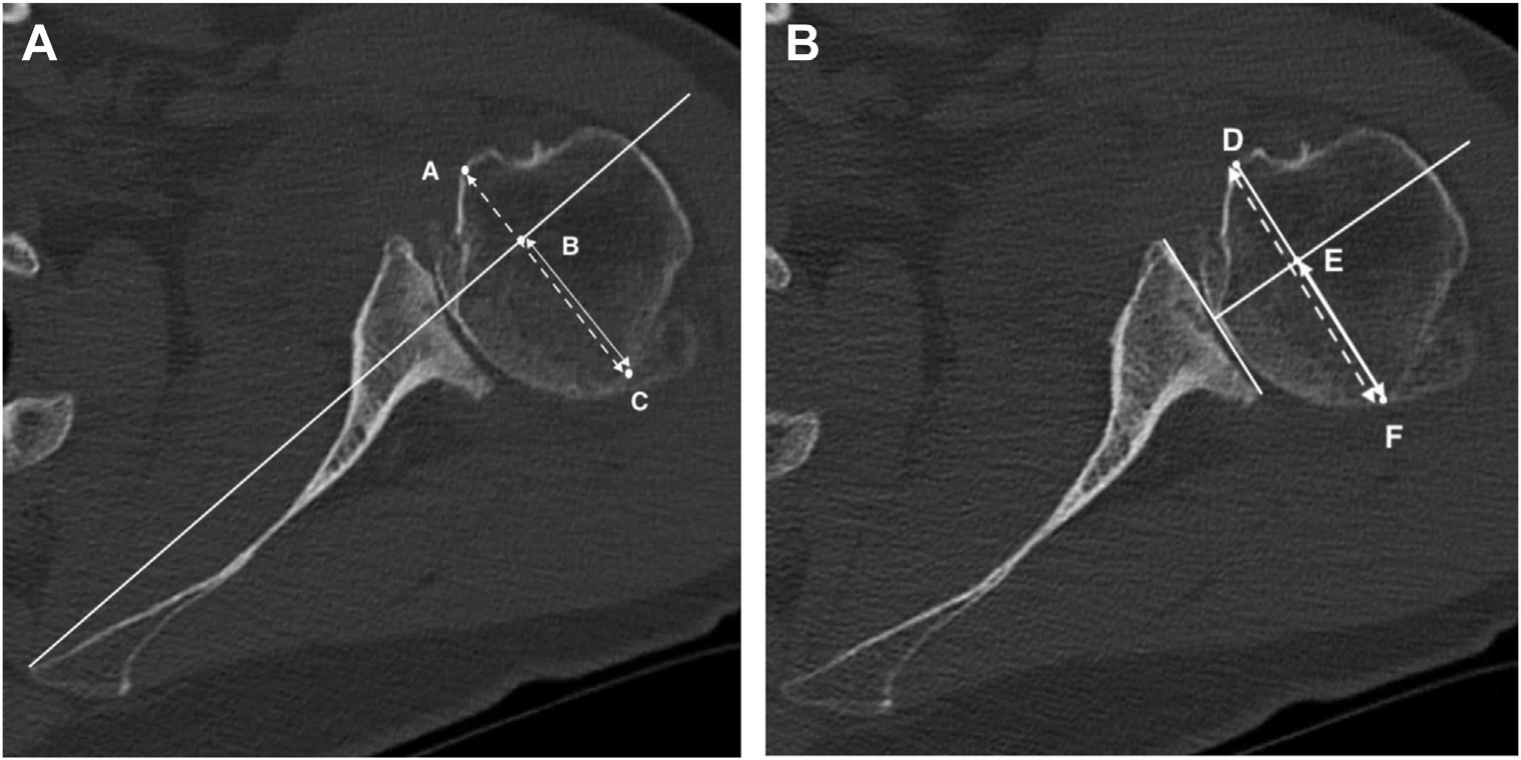
Humeral head subluxation measurements. **(A)** The humeral head subluxation was measured relative to the axis of the scapula or Friedman line by establishing a perpendicular line from the axis of the scapula and measuring the length BC divided by the length AC. **(B)** The humeral head subluxation was measured relative to the axis of the intermediate glenoid or the mediatrice method by establishing a perpendicular line from the center of the intermediate glenoid line and measuring the length EF divided by the length DF.

Preoperative imaging for all patients included true anteroposterior, axillary lateral, and scapular lateral shoulder radiographs and a native shoulder CT scan. All CT scans for the follow-up study were performed on a brilliance 64-channel CT scanner (SOMATOM Definition AS; Siemens, Munich, Germany). The grade of osteoarthritis, classified by Samilson^25^ and the acromiohumeral distance (ACHD) were both analyzed on a true anteroposterior radiographs.^4^ The glenoid inclination was measured on the shoulder CT scan.^17^ Glenoid retroversion measurement was made according to the Friedman technique^5^ and to consider the special morphology of B2 glenoid, the retroversion of the intermediate glenoid, the paleoglenoid and the neoglenoid were taken (Fig. 2, *A* and *B*).^23^^,^^29^ Measurement of the posterior wear was measured in depth (Fig. 2*B*).^29^ The humeral head subluxation was measured both relative to the axis of the scapula and also relative to the axis of the intermediate glenoid or the mediatrice method (Fig. 3, *A* and *B*).^28^^,^^29^

Postoperative imaging included true anteroposterior, axillary lateral, and scapular lateral shoulder radiographs. The radiolucency lines around the humeral and glenoid component were analyzed according to the Lazarus classification.^15^ When postoperative shoulder CT scans were available, the mean glenoid retroversion, humeral head subluxation, and glenoid radiolucency were analyzed according to the technique of Yian et al.^31^ The glenoid component was defined to be loose if there was an obvious subsidence, if the radiographic Lazarus score was ≥4 or if the loosening on the shoulder CT scan was ≥6 according to the modified Molé scores.^15^^,^^31^

All measurements were made independently by 2 authors (A.H., S.H.) who were blinded to each other results and the average measurements are reported.

### Statistical analysis

The Shapiro-Wilk test was applied to test the data for normal distribution. Categorical variables were tested by the Chi-squared and Fisher’s exact test, respectively. Preoperative and postoperative radiographic analyses and functional scores were compared using the paired t test (normal data) and the Wilcoxon signed-ranks test (non-normal data). The Mann-Whitney *U* test was applied for subgroup analysis. The interobserver reliability of the assessments of the glenoid retroversion, humeral head subluxation, radiolucency scores were measured by calculating the intraclass correlation coefficient (ICC) for absolute agreement, with 1 indicating perfect reliability. Kaplan–Meier curve analysis was used to analyze the implant survivorship of all potential cases including the 15 patients who died or were lost to follow-up and 95% confidence intervals (CIs) were calculated. The Pearson correlation coefficient was calculated to assess bivariate correlation between glenoid loosening assessed by plain radiographs and by CT scans. The significance level was set at 0.05 and all *P* values were 2-tailed.

## Results

### Imaging outcome

The preoperative radiological measurements are reported in Table II. Of the 32 shoulders, 3 patients (9.4%) had a neoglenoid retroversion >27° and the mean glenoid wear was −3 mm (range, −1 to −6 mm, ±1). At the time of the latest follow-up, radiographs were available for all 32 shoulders at a mean of 8.9 years (range, 1.0-16.6 years, ±3.2) follow-up. Only one patient had a shoulder radiograph done 1 year postoperative, but he had a postoperative shoulder CT scan done at 5.0 years follow-up. Glenoid loosening was evaluated with the Lazarus classification on the final available shoulder radiograph and when available, with the shoulder CT scan according to the modified Molé scores, and their analyses are available in Table III. To control for potential cofounding variables, we compared the demographic data and preoperative radiological analysis of these 2 groups (not loose vs. loose). There was no significant difference in sex, body mass index, dominance, primary procedure or previous non-arthroplasty procedure, neoglenoid retroversion >27°, or humeral subluxation >65% according to the plane of the scapula and to the plane of the glenoid between the 2 groups (*P* > .05). However, when the absolute Lazarus score was higher, there was a statistically significant difference in postoperative aCS (*P* < .001), rCS (*P* = .001), SSV (*P* = .049), forward active elevation (*P* = .020), internal rotation (IR) (*P* = .007), absolute strength (*P* = .032), relative strength (*P* = .037), and satisfaction (*P* = .022). When comparing shoulders that had preoperative intermediate glenoid retroversion <20° (n = 25) with those that had ≥20° intermediate glenoid retroversion (n = 7), there was no statistically significant difference in the last follow-up Lazarus score (*P* = .395).

**Table II:** Preoperative radiological measurements (n = 32 shoulders).

Variable	Mean (range)	Standard deviation
Acromiohumeral distance (mm)	9 (5-14)	2
Samilson grade	4 (3-4)	0
Glenoid inclination (°)	76 (55-89)	7
Intermediate glenoid version (°)	−14 (−2 to −29)	7
Paleoglenoid version (°)	−13 (−1 to −27)	6
Neoglenoid version (°)	−13 (1 to −34)	8
Glenoid wear (mm)	−3 (−1 to −6)	1
Humeral subluxation (scapula axis method) (%)	67 (49-87)	9
Humeral subluxation (mediatrice method) (%)	56 (48-69)	5

.

**Table III:** Glenoid radiolucency measurements on shoulder radiograph at mean 8.9 years (range, 1.0-16.6 years, ±3.2) follow-up (n = 32) and on postoperative shoulder CT scan at mean 5.0 years (range, 0.1-10.3 ± 3.0) follow-up (n = 22).

Not loose			Loose		
Lazarus (n = 32 shoulders)	Grade 0	N = 8	Lazarus (n = 32 shoulders)	Grade 4	N = 1
	Grade 1	N = 10		Grade 5	N = 5
	Grade 2	N = 6			
	Grade 3	N = 2			
Modified Molé scores (n = 22 shoulders)	Score 0 point	N = 6	Modified Molé scores (n = 22 shoulders)	Score 6 points	N = 0
	Score 1 point	N = 5		Score 7 points	N = 1
	Score 2 points	N = 5		Score 8 points	N = 0
	Score 3 points	N = 1		Score 9 points	N = 0
	Score 4 points	N = 0		Score 10 points	N = 0
	Score 5 points	N = 2		Score 11 points	N = 0
				Score 12 points	N = 2

*CT*, computed tomography.

A postoperative shoulder CT scan was obtained in 22 shoulders at a mean of 5.0 years (range, 0.1-10.3, ±3.0) follow-up and measurements are reported in Table IV. Thirteen of 22 patients had a preoperative posterior humeral head subluxation ≥65% and posterior static humeral head subluxation was reversed in 11 of 13 patients after surgery. There was no significant correlation between amount of correction and pain, and any of the CS variables (*P* > .05). However, when divided into correction to <10° of retroversion (n = 19) vs. the undercorrected/not corrected >10° of glenoid retroversion (n = 3), patients with corrected glenoid retroversion revealed significantly better active IR (mean 9 IR CS points) compared with patients with undercorrected/not corrected glenoid retroverion (mean 8 IR CS points; *P* = .035). There were no significant correlations between preoperative subluxation and loosening based on postoperative CT scans available for the 22 patients (*P* = .529). Postoperatively, CT scan showed glenoid radiolucencies in 16 of 22 cases available (76.2%) with a mean radiolucency score of 3.0 points (range, 0-12). There was a highly significant correlation between glenoid loosening assessed by plain radiographs and by CT scans (r = 0.77; *P* < .001). In 3 patients (14.3%) a score of ≥6 identifying loosening of the glenoid were documented and 2 patients underwent revision. There were 2 patients with shaft loosening and one of them required revision.

**Table IV:** Radiological measurements on preoperative and postoperative shoulder CT scan at a mean follow-up of 5.0 years (range, 0.1-10.3 ± 3.0), (n = 22).

Variable	Preoperative	Postoperative	*P* value
Intermediate glenoid retroversion (°)	−14 (−2 to −23) ± 6	−5 (6 to −28) ± 8	<.001
Humeral head subluxation (scapula axis method) (%)	67 (49-87) ± 9	55 (44-70) ± 7	<.001
Humeral head subluxation (mediatrice method) (%)	56 (48-69) ± 6	53 (44-70) ± 6	.196

*CT*, computed tomography.

### Complications and reoperations

In total, 5 complications were identified in 5 of 30 patients (32 shoulders) (15.6%) (1 instability, 2 infections, 1 aseptic loosening, and 1 revision for subscapularis) and 4 of these 5 complications were operated and required revision (12.5%). One patient was revised because of nonunion of the lesser tuberosity osteotomy and had a good function at last follow-up of 11.3 year (rCS: 88.48%, aCS 74 points, and SSV: 90%). The 4 other complications (1 instability, 2 infections, and 1 aseptic loosening) were excluded from clinical analysis, but included in the Kaplan–Meier analysis for survivorship without implant revision of the TSA. Three patients (9.4%) required a revision surgery with removal of the primary implant (1 instability, 1 infection, and 1 aseptic loosening). One patient had a posterior shoulder instability and first underwent an arthroscopic posterior capsular shift 10 months after the initial TSA (Lazarus Grade 0) and 87 months later, the aTSA was revised to a rTSA because of ongoing instability (Lazarus Grade 0). One patient was diagnosed with a late periprosthetic infection 4.6 years after the initial surgery (Lazarus Grade 2) and had a staged revision and conversion to a hemiarthroplasty. One patient had a conversion to rTSA for persistent pain and aseptic loosening (Lazarus Grade 4) 4 years after the initial surgery. One patient had a possible infection with loosening of the glenoid (Lazarus Grade 5) 11 years after the implantation. This patient died at the age of 81 years unrelated to the shoulder problem before the revision could be performed (excluded from clinical analysis).

Failure was not statistically associated with preoperative body mass index (*P* = 1.000), gender (*P* = .603), dominance (*P* = 1.000), previous surgery (*P* = .150), neoglenoid retroversion >27° (*P* = 1.000), or humeral subluxation > 65% according to the axis of the scapula and humeral head subluxation according to the axis of the intermediate glenoid (*P* > .05).

### Clinical outcome

There was a significant improvement (all *P* = .000) for all the postoperative clinical outcome parameters as detailed in Table V. Regarding the subjective results at the last follow-up available, 81.3% of patients were very satisfied, 15.6% were satisfied, 3.1% were uncertain, and no patient was disappointed with the results. There was no significant association between preoperative radiologic evaluation of paleoglenoid version, intermediate glenoid version, neoglenoid version, posterior wear, humeral head subluxation, and postoperative clinical outcome of aCS, rCS, SSV, forward active elevation, abduction, external rotation, and IR.

**Table V:** Clinical findings preoperatively and final follow-up [mean 9.9 years (range, 5.0-16.6, ±2.8)] (n = 28).

Variable	Pre-op	Latest follow-up	*P* value (pre-op. vs. latest follow-up)
CS			
Absolute (points)	42 ± 17	78 ± 7	<.001
Relative (%)	52 ± 19	94 ± 7	<.001
Pain (points)	5 ± 3	14 ± 2	<.001
Strength (points)	5 ± 4	11 ± 4	<.001
Strength (%)	6 ± 5	14 ± 4	<.001
SSV (%)	40 ± 16	90 ± 11	<.001
Range of motion			
Active anterior elevation (°)	104 ± 32	151 ± 20	<.001
External rotation (°)	21 ± 18	45 ± 17	<.001
Internal rotation (points)	6 ± 2	9 ± 1	<.001
Abduction (°)	88 ± 34	152 ± 23	<.001

*CS*, clinical score; *Preop,* preoperative; *SSV*, subjective shoulder value.

### Survival analysis

**Figure 4:**
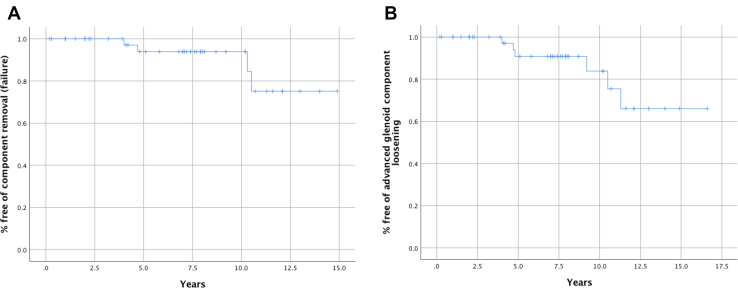
Kaplan–Meier survivorship analysis. **(A)** Survivorship without implant revision (years). **(B)** The rate of radiographically stable glenoid components*.*

Based on Kaplan–Meier analysis, the estimated survivorship without implant revision of the TSA was 94% at 5 years and 85% at 10 years (12.1-14.7 years) (Fig. 4*A*). The survival rate without advanced glenoid component loosening (defined as Lazarus grade ≥ 4 or modified Molé scores ≥ 6) was 91% at 5 years and 84% at 10 years (12.2-15.8 years) (Fig. 4*B*).

## Discussion

In the present study, a revision rate of 12.5% and a complication rate of 15.6% were observed at a mean follow-up of 9.2 years (range, 5.0-16.6, ±3.2). The estimated survivorship without implant revision of the TSA was 85% at 10 years (12.1-14.7 years) but more importantly, the survival rate without advanced glenoid component loosening (defined as Lazarus grade ≥ 4 or modified Molé scores ≥ 6) was 84% at 10 years (12.2-15.8 years). Six patients (18.8%) presented with signs of glenoid loosening. In this study, the final Lazarus score was not correlated with preoperative intermediate glenoid retroversion <20° or ≥20° (*P* = .395) or preoperative paleoglenoid version, neoglenoid version, posterior wear, humeral head subluxation, and postoperative clinical outcomes. In the Australian Orthopaedic Association National Joint Replacement Registry (AOANJRR) 2021 annual report, the cumulative percent of revision of primary total stemmed shoulder replacement for primary osteoarthritis was 7.2 (6.6, 7.8) at 5 years and 11.7 (10.7, 12.9) at 10 years.^1^ The revision rate in our case series with mild B2 deformity treated with asymmetric reaming and TSA is close to the cumulative percent of revision of primary total stemmed shoulder replacement for primary shoulder osteoarthritis reported in the AOANJRR 2021 report. These outcome results refute the main hypothesis of this study, namely that cementation of a glenoid component in B2 glenoids has a poor radiographic outcome and a high, early revision rate. Although, in this study, advanced radiographic loosening is associated with an inferior clinical result.

These findings are contrasting the results reported by Walch et al^29^ who reported a high early complication and failure rate of aTSA in B2 glenoids. At a mean follow-up of 77 months, they reported 20.6% of glenoid loosening and a rate of revision of 16.3% (6.5% of revisions were for glenoid loosening, 5.5% for posterior instability, and 4.3% for soft tissue problems) in 92 aTSA (75 patients). The mean preoperative intermediate glenoid version was 19.2°, the mean posterior wear was 7.3 mm, and the mean humeral subluxation referenced to the scapula was 73.1%. A 50% rate of complication was found when the intermediate retroversion was over 30° and 44% complication rate when the retroversion of the neoglenoid was over 27°. The difference in results found in our case study can be partly explained by the different population with a milder form of B2 deformity where the mean preoperative intermediate glenoid version was −14°, the mean posterior glenoid wear was −3 mm, and the mean humeral subluxation was 66%. Also, there were only 3 patients who had a preoperative neoglenoid retroversion >27° and no patient had an intermediate glenoid retroversion ≥30°.

The observed clinical improvement and relatively low rate of radiological radiolucency in this study are consistent with Orvets et al^19^ who reported good short-term results of 59 aTSA with partial corrective reaming in B2 glenoid at a mean follow-up of 50 months. The mean preoperative intermediate glenoid retroversion was 18° and the mean posterior humeral subluxation was 67%. There was no case of revision for glenoid loosening or instability and significant improvement (*P* < .001) of clinical outcome with final visual analog scale pain score of 1.4, mean American Shoulder and Elbow Surgeons score of 84.3, and STT of 9.1. Also, no difference in progression of glenoid radiololucency between preoperative intermediate glenoid retroversion ≤20° (27.8%) compared to preoperative intermediate glenoid retroversion >20° (22.7%, *P* = .670) was found. Also, Sheth et al^26^ reported good short-term results at a mean follow-up of 40 ± 15 months of aTSA for the treatment of 111 preoperative Walch B2 glenoid and 178 preoperative Walch A1 glenoid. Preoperative shoulder CT scan images were available for only 61% of the preoperative Walch B2 glenoid, the mean glenoid retroversion was 15 ± 6°, and no data were provided for the posterior humeral subluxation. There was no significant difference at final follow-up between Walch B2 and A1 glenoid in terms of clinical scores (CS subscales pain-activity-mobility-adjusted, American Shoulder and Elbow Surgeon Standardized Shoulder Assessment Form, Single Assessment Numerical Evaluation, and patient satisfaction), revision rates, complication rates, and glenoid radiolucencies. The CS subscale strength and the total CS at the final follow-up were both significantly better in B2 glenoid group. Three patients had a posterior shoulder dislocation in the preoperative Walch B2 group and all required revision.

A recent systematic review on outcomes of aTSA with B2 glenoids reported a total of 239 shoulders, mean age of patients 63.3 years (range, 55.8-68.7 years) with a mean follow-up of 55.5 months (range, 24-91 months) collected from 10 different studies.^2^^,^^6^^,^^11^^,^^14^^,^^16^^,^^22^^,^^24^^,^^27^^,^^29^^,^^30^ The majority of patients reported were treated with asymmetric reaming technique that was performed in 127 TSA and the preoperative diagnostic of B2 glenoid was made base on a preoperative shoulder CT scan for these patients.^2^^,^^6^^,^^29^ Posterior bone grafting was performed in 73 shoulders;^11^^,^^14^^,^^24^^,^^27^ however, the preoperative diagnosis of B2 glenoid was based on preoperative radiographic evaluation in 64 of 73 shoulders.^11^^,^^14^^,^^27^ Finally, 34 shoulders ^22^^,^^30^ had an augmented glenoid component for correction of bone loss and glenoid retroversion where the diagnostic of preoperative B2 glenoid was made on shoulder radiographs in 14 of 34 cases.^22^ The CSs were similar for the 3 surgical techniques (CS 73 points for asymmetric reaming, CS 79 points for posterior bone-grafting, and CS 75 points for posteriorly augmented glenoid). The rate of revision was 15.6% for asymmetric reaming, 9.5% for posterior bone-grafting technique, and no revision in the posteriorly augmented glenoid implant. The most common complication was glenoid radiolucent lines. In this systematic review, the lack of preoperative shoulder CT scan in 78 of 239 shoulders to accurately qualify and quantify the severity of preoperative glenoid deformity is raising the concern about the validity of extrapolation of these results nowadays and reflects the lack of information about this topic.

The preoperative posterior humeral head subluxation in B2 glenoids is a concerning factor to consider as the eccentric loading leads to an increased risk of glenoid loosening. Glenoid loosening is the most important reason of long-term failure of TSA.^10^^,^^12^^,^^15^^,^^20^ In the current study, 22 patients had a postoperative shoulder CT scan and 13 of 22 had a preoperative posterior humeral head subluxation ≥65%. The preoperative glenoid retroversion was restored to a mean intermediate glenoid version of −5° (6° to −28°, ±8), humeral head subluxation to the scapula axis was 55% (range, 44%-70%, ±7), and the posterior humeral head subluxation (≥65%) was reversed in 11 of 13 patients. There was no significant correlation between the amount of correction and pain, and any of the CS variables (*P* > .05). However, the statistical difference found in active IR between glenoid retroversion correction to <10° (n = 19) (mean 9 IR CS points) vs. the undercorrected/not corrected >10° of glenoid retroversion (n = 3) (mean 8 IR CS points; *P* = .035) needs to be interpreted with caution as the sample size is likely to be underpowered. Similarly, Gerber et al^6^ reported 23 cases of shoulders (Walch type B1 in 9 patients, type B2 in 5, and type C in 9) with primary TSA that had static preoperative posterior humeral head subluxation ≥65% on shoulder CT scan and a mean glenoid retroversion of 18° (range, 0° to −40°). After surgery, the posterior static humeral head subluxation was reversed in 21 of 23 patients with an average humeral head subluxation of 50% (range, 40%-68%) and an average glenoid retroversion of 9° (range, 0° to −25°).

The strengths of our study are that it is a monocentric, mid- to long-term follow-up of aTSA in B2 glenoid where all patients had a preoperative shoulder CT scan. To our knowledge, this is the longest follow-up reported for clinical and radiological results of aTSA and asymmetric reaming in B2 glenoid. Limitations of this study include the retrospective noncomparative design and the relatively small number of patients that could lead to a type I error. Overall, 15 patients (31.9%) were lost to follow-up, where 12 patients (25.5%) were explained by unrelated death before final follow-up. Also, a postoperative shoulder CT scan was not available for all cases and radiolucency could have been underestimated in shoulder radiographs.^31^

## Conclusion

In this case series, the outcome results refute the main hypothesis of this study, namely that cementation of a glenoid component in B2 glenoids has a poor radiographic outcome and a high, early revision rate. aTSA with asymmetric reaming for the treatment of shoulder osteoarthritis with milder forms of B2 glenoid is a viable option with good mid- to long-term clinical and radiological results and a lower than expected failure rate.

## Disclaimers

Funding: No funding was disclosed by the authors.

Conflicts of interest: Dr Christian Gerber receives royalties from Zimmer-Biomet which is related to the subject of this work. Dr Karl Wieser works as a consultant for Zimmer-Biomet. The other authors, their immediate families, and any research foundation with which they are affiliated have not received any financial payments or other benefits from any commercial entity related to the subject of this article.
